# Workforce Implications of Increased Referrals to Hereditary Cancer Services in Canada: A Scenario-Based Analysis

**DOI:** 10.3390/curroncol30080525

**Published:** 2023-07-29

**Authors:** Nick Dragojlovic, Kennedy Borle, Nicola Kopac, Amy Nisselle, Jennifer Nuk, Mandy Jevon, Jan M. Friedman, Alison M. Elliott, Larry D. Lynd

**Affiliations:** 1 Collaboration for Outcomes Research and Evaluation, Faculty of Pharmaceutical Sciences, University of British Columbia, Vancouver, BC V6T 1Z3, Canada; nick.dragojlovic@ubc.ca (N.D.);; 2 Australian Genomics Health Alliance, Parkville, VIC 3052, Australia; 3 Murdoch Children’s Research Institute, Department of Pediatrics, The University of Melbourne, Parkville, VIC 3052, Australia; 4 Hereditary Cancer Program, BC Cancer, Vancouver, BC V5Z 1J2, Canada; 5 Department of Medical Genetics, Faculty of Medicine, University of British Columbia, Vancouver, BC V6H 3N1, Canada; 6 BC Children’s Hospital Research Institute, Vancouver, BC V5Z 4H4, Canada; 7 Women’s Health Research Institute, Vancouver, BC V6H 3N1, Canada; 8 Centre for Health Evaluation & Outcome Sciences, Providence Health, Vancouver, BC V6Z IY6, Canada

**Keywords:** workforce planning, health human resource planning, genetic counselling, genetic testing, genomics, clinical genetic services, needs-based planning

## Abstract

Over the last decade, utilization of clinical genetics services has grown rapidly, putting increasing pressure on the workforce available to deliver genetic healthcare. To highlight the policy challenges facing Canadian health systems, a needs-based workforce requirements model was developed to determine the number of Canadian patients in 2030 for whom an assessment of hereditary cancer risk would be indicated according to current standards and the numbers of genetic counsellors, clinical geneticists and other physicians with expertise in genetics needed to provide care under a diverse set of scenarios. Our model projects that by 2030, a total of 90 specialist physicians and 326 genetic counsellors (1.7-fold and 1.6-fold increases from 2020, respectively) will be required to provide Canadians with indicated hereditary cancer services if current growth trends and care models remain unchanged. However, if the expansion in eligibility for hereditary cancer assessment accelerates, the need for healthcare providers with expertise in genetics would increase dramatically unless alternative care models are widely adopted. Increasing capacity through service delivery innovation, as well as mainstreaming of cancer genetics care, will be critical to Canadian health systems’ ability to meet this challenge.

## 1. Introduction

Hereditary cancer syndromes are caused by heritable genetic variants in tumor suppressor genes or proto-oncogenes and account for 5–10% of all cases of cancer in North America [1]. Genetic testing and genetic counselling for hereditary cancer syndromes can improve outcomes for patients and their at-risk relatives [2,3] by informing cancer surveillance, prevention, screening, preventative strategies, and treatment as well as reproductive decision-making. A number of factors have driven a rapid increase in the utilization of cancer genetics services over the last fifteen years in Canada and other high-income countries [4,5], including increased awareness among patients and providers and an expansion in the eligibility criteria for genetic assessment, as knowledge about the natural history, specific genetic risk factors and types of cancer associated with hereditary cancer syndromes has grown over time. For example, the number of patients referred to Ontario genetics clinics for cancer indications nearly tripled between 2007 and 2016 [4]. In contrast, the number of genetic counsellors (GCs) serving that patient population only increased by 26% and the number of genetics clinic physicians actually declined [4]. The average wait time for non-urgent cancer referrals to these clinics was 183 days [4].

The Canadian clinical genetics workforce as a whole, which includes GCs as well as clinical geneticists and other specialist physicians with expertise in genetics (genetic medical doctors or GMDs), is facing significant pressure as a result of this rapid growth in utilization. While it currently includes approximately 110 clinical geneticists and 485 GCs [6,7,8], about 35% of whom practice primarily in the area of cancer care [9,10], average wait times for non-urgent genetics appointments of all kinds in Canada are between 6 and 12 months [4,9] with some clinics reporting average waits of up to 20 months. Cancer Care Ontario estimated that in 2018 it only had about half of the GC capacity necessary to meet the need for hereditary cancer services in the province [4]. This gap is likely to expand as the need increases.

To explore this nation-wide challenge and inform strategic planning for cancer genetics healthcare in Canada, we estimated the number of GCs and GMDs that will be required to meet the projected need for hereditary cancer services in 2030 under a range of scenarios.

## 2. Materials and Methods

We constructed a workforce requirements model using the needs-based approach to health human resources planning (Birch et al. [11]—see the Supplementary Materials for full details). The model estimates the number of GMDs and GCs required to meet the need for hereditary cancer services in Canada in 2020 and the projected future need in 2030, with “need” defined as the capacity to benefit from services [12]. Input parameters include demographic and epidemiological (i.e., incidence of cancer) characteristics of the Canadian population, estimates of the appropriate level of service (e.g., eligibility for genetic testing and counselling) for different patient sub-populations, and the productivity of the workforce (Table S1).

**Figure 1:**
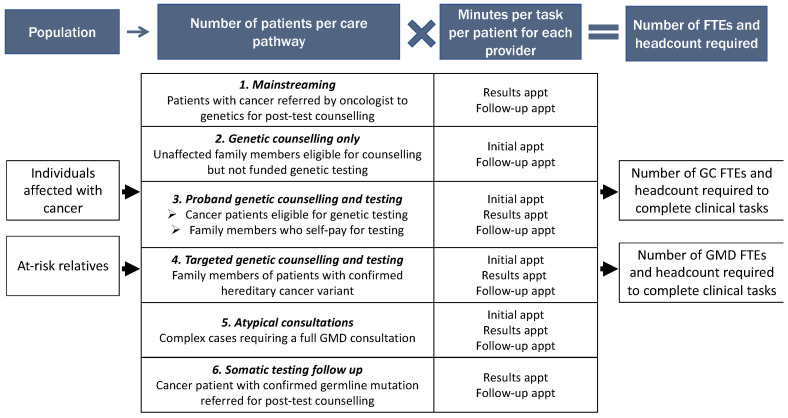
Describes the structure of the needs-based hereditary cancer workforce requirements model, including six clinical pathways providing different levels of service to specific patient sub-populations (Table S2). FTEs: Full-time equivalents; GC: Genetic counsellor; GMD: Medical doctor with expertise in genetics.

Six clinical care pathways for hereditary cancer services were mapped out, based on collaboration with content experts, the authors’ clinical experience, and the literature (see Figure 1). In Pathways #1–4 and #6, hereditary cancer appointments are assumed to be primarily led by the GC, with the GMD involved on a supervisory basis to review the case, provide input on management, order genetic testing, review and sign the clinical letter, and provide clinical oversight. In contrast, Pathway #5 (“atypical consultations”) captures patients with more complex presentations (including syndromes with extra-oncologic features and pediatric cases) who require a full GMD consultation and for whom a GMD takes the lead in providing care for the patient. The annual number of patients seen through each pathway in a given jurisdiction was estimated based on the incidence of different types of cancer, clinical guidelines, and a range of parameters drawn from the literature that aim to capture referral patterns and patient uptake (Tables S2 and S3).

Provider time required for each of five service units (triage, initial appointment, test coordination, results appointment, and additional follow-ups; Table S4) was estimated by adapting data from an Australian workforce survey to the Canadian context [13]. The total provider time required to serve the projected number of patients in all clinical pathways was then converted into direct patient care full-time equivalents (DPC-FTEs), a workforce metric that assumes that all healthcare providers allocate 100% of their time to direct patient care (1 DPC-FTE = 1800 h of direct patient-related activities per year). These were then converted into an estimated headcount (i.e., the number of individual providers) by accounting for time devoted to tasks not directly related to patient care (e.g., administration, teaching, research and professional development), variation in average hours worked per week, and part-time employment (Supplementary Materials Section 3.2).

The model base case linearly extrapolates historical growth in the rate of genetics referrals for newly diagnosed cancer patients to account for continued eligibility expansion in clinical guidelines (Table S3). However, a recent Delphi panel suggested that this trend may in fact accelerate over the next decade [14]. Participants estimated that 32% of cancer patients would receive germline genetic testing by 2030 (substantially above the referral rates used in the base case, i.e., 9% in 2020 and 14% in 2030, which were defined using country-wide historical utilization data from France [15]). Moreover, they also expected an increase in the use of genome-wide sequencing (GWS; exome or genome sequencing), which due to its greater complexity, calls for genetic counselling in all cases [16] and requires more provider time [13]. We therefore also created hypothetical scenarios to understand the impact of potential future changes in referral volume, testing technology and service delivery models on estimated workforce requirements (Table S5).

Finally, we conducted one-way deterministic and probabilistic sensitivity analyses (DSA and PSA, respectively) to characterize and explore uncertainty in the model’s projections. The DSA varied each parameter by ± 25% while holding other variables constant (Supplementary Materials, Section S5), while the PSA consisted of 1000 iterations of a Monte-Carlo simulation in which parameter values were randomly sampled from distributions defined as shown in Table S3 to estimate the effect of stochastic variation.

## 3. Results

The base case model estimated that in 2020 there was a need for 53 physicians with expertise in genetics (95% CI: 41, 70) and 199 genetic counsellors (95% CI: 135, 287) to provide hereditary cancer services in Canada (Table 1). There were actually 378 [17] GCs working clinically in Canada in 2020—with about 35% of GCs specializing in cancer genetics [9,10]. In 2019, there were 111 clinical geneticists in Canada [7], but some GMDs in cancer genetics clinics are not clinical geneticists but oncologists with a particular expertise in hereditary cancer. The projected workforce needed in 2030 was 91 GMDs (95% CI: 71, 117) and 329 GCs (95% CI: 228, 465), representing increases of 71% and 65%, respectively, relative to 2020.

In our deterministic sensitivity analysis in which a set of 20 inputs were varied by +/−25%, six factors had the largest impact on workforce requirements for both GMDs and GCs (Figures S1 and S2). Three of these related to services for unaffected family members: the number of first-degree relatives per cancer case, the proportion of these who are designated as high-risk by their primary healthcare providers, and the proportion of family members informed of a direct relative’s confirmed pathogenic variant who pursue a referral for cascade testing. The other three factors were the proportion of incident cancer patients referred to hereditary cancer services and the time spent in pre- and post-test appointments for encounters with GCs and for GMDs under the various care pathways (see Table S3). Notably, the time spent per appointment by GMDs when providing a full consultation and the percent of accepted referrals of new cancer patients that are classified as atypical (Pathway 5, Figure 1) were secondary drivers of overall GMD workforce requirements in the sensitivity analysis, due to the relatively small proportion of patients in this category.

### Scenarios

**Figure 2:**
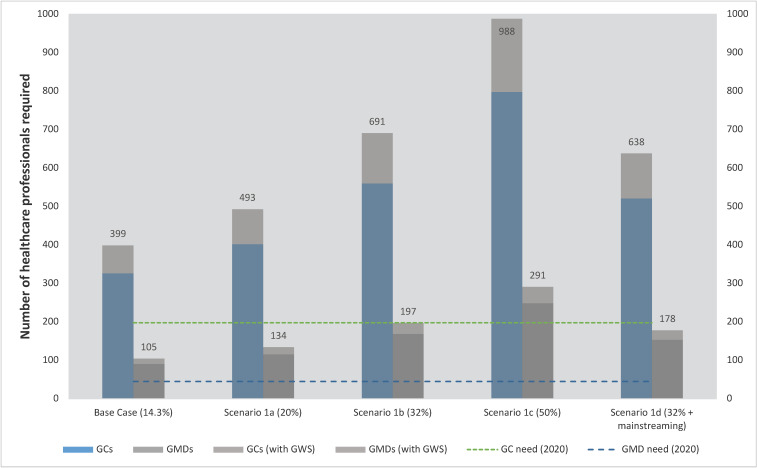
Projected need for Canadian cancer genetics providers in 2030, by scenario. This figure shows the effect of increased volume of patients on the number of genetics healthcare providers required in 2030, and then further effects when panel testing is replaced by genome wide sequencing and increased mainstreaming. Scenario values derived from Delphi study estimates [14] (see Table S5). GC; genetic counsellor, GMD; medical doctor with expertise in genetics, and GWS; genome-wide sequencing (genome or exome sequencing).

Figure 2 shows the impact of increasing the proportion of newly diagnosed cancer patients who will be referred for a hereditary cancer assessment in 2030 from 14.3% in our base case to the 32% expected by Canadian genetics experts [14]. In addition, it shows the impact of substituting all gene panel tests with genome-wide sequencing (GWS) and of partially mainstreaming all breast cancer patients (with non-GMD physicians ordering germline genetic tests and only patients with confirmed cancer risk variants being referred to genetics). Increasing the expected referral rate from 14.3% to the 32% predicted by the Delphi panel (Scenario 1b) resulted in a 72% and 88% increase in the headcount required for GCs and GMDs, respectively, while GWS substitution on its own only increased workforce requirements by 22% and 17%, respectively. Mainstreaming all breast cancer patients in a scenario where the referral rate increases to 32% (Scenario 1d) reduced the projected GMD and GC workforce requirements by 9.5% and 7%, respectively, but these still represent increases of 70% and 60% relative to the base case.

**Figure 3:**
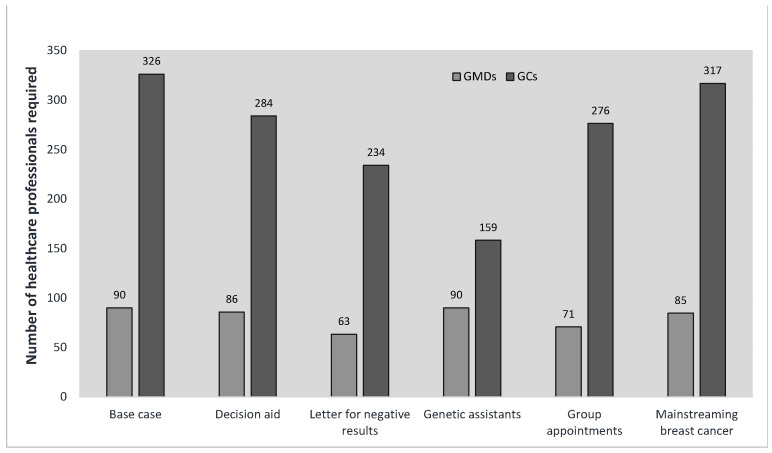
The impact of alternative service delivery models on workforce requirements. Shows the impact of innovations in the hereditary cancer service delivery model on the number of genetics healthcare providers required in 2030 (see Table S5 for details on how scenarios are defined). GC; genetic counsellor, and GMD; medical doctor with expertise in genetics.

In contrast, Figure 3 shows the estimated impact of five possible changes to the current standard service delivery model. Using a letter to return negative test results instead of a GC/GMD appointment had a large impact on workforce requirements for both provider types, reducing necessary FTEs by 28% for GCs and 30% for GMDs relative to the base case, as did using a group appointment model for some types of pre-test counseling. In contrast, using a self-administered digital decision aid to substitute for most pre-test genetic counselling and introducing genetic assistants into a clinic to support administrative tasks like test coordination currently performed by GCs primarily impacted the GC FTE requirements (13% and 51% reductions, respectively). Mainstreaming all breast cancer patients only had a modest impact on GMD and GC workforce requirements given the 14% referral rate in the base case.

## 4. Discussion

Our model suggests that under existing trends, referrals to hereditary cancer services in Canada will likely increase by more than 50% from 2020 to 2030. Concomitantly, a total of 91 GMDs (95% CI 71, 117) and 329 GCs (95% CI 228, 465) will be required to meet the projected need in 2030 (Table 1). The sensitivity analysis suggests that in addition to patient referral rate, provider times allocated per service unit and the number of unaffected family members of cancer patients receiving hereditary cancer assessment were the most significant drivers of human resource requirements (Figures S1 and S2). In scenario analyses, accelerated expansion in the eligibility of cancer patients for hereditary cancer assessments would increase the estimated 2030 workforce requirements far beyond the base case estimates (Figure 2). Replacing gene panels with GWS would have a smaller, though still important, impact (Figure 2), because GCs and GMDs report significantly more time required per case when GWS is used [18] due to the additional counseling and interpretation necessary for GWS [16,18]. However, while wholesale replacement of gene panels for germline testing of cancer patients with GWS was deemed likely by only 55% of Canadian genetics experts in our Delphi study [14], the present findings have broad applicability. Specifically, one reason for the increased complexity in counseling and interpretation for GWS is the higher prevalence of variants of unknown significance (VUS). However, the growth in the use of large gene panels that are themselves associated with a higher prevalence of VUS [19] suggests that the GC and GMD time per case may increase regardless. Finally, we find that a range of service delivery innovations could reduce workforce requirements by making it more efficient for GMDs and GCs to provide care for more patients (Figure 3).

The Canadian clinical genetics workforce in 2020 for all clinical areas was estimated to include 111 clinical geneticists [7], a small number of oncologists and other non-geneticists clinically focused on genetic diseases, and 378 clinically practicing GCs [17]. Thus, our model’s 2020 estimated need for 53 GMDs and 200 GCs working in direct patient care in cancer represents approximately 50% of the available genetics workforce. This is consistent with other studies that have shown that cancer accounts for a large number of patients seen in genetics clinics [4] and is the most common practice area for new GCs [6,20]. However, considering that there are only about 20–25 graduates from Canadian genetic counseling programs per year [21], with only about 10–12 choosing a cancer specialty, Canadian genetics clinics may struggle to meet the base case’s projected need for an additional 129 GCs by 2030.

In 2018, Cancer Care Ontario also performed a needs-based analysis that considered both projected change in the incidence of new cancers by primary site and the estimated proportion of patients for whom a genetics assessment would be clinically indicated [4], though the approach and input data differed from our model. Nonetheless, it estimated a very similar level of need (25,810 necessary referrals for 2016, only 10% below our model’s estimate; Table S6), lending face validity to our results. Our study takes the needs-based approach further by expanding the analysis to Canada as a whole, systematically exploring uncertainty in our projections using sensitivity and scenario analyses, and accounting for the highly dynamic nature of genetic medicine. For example, the compound annual growth rate in the need for GCs projected by Cancer Care Ontario’s model (2.3% per year) was only about half as large as our model (5.1% per year), which we believe results from it not accounting for continued growth in the proportion of new cancer patients eligible for genetic evaluation, a prospect that Canadian genetics experts deemed highly likely in the Delphi panel study [14].

In addition to the expected growth in eligibility for hereditary cancer services, it is also worth considering the implications of a potential acceleration in the move towards integrating hereditary cancer genetics into oncology clinics. On the one hand, commercial next generation sequencing (NGS)-based tumour testing panels often include numerous genes linked to hereditary cancer [22] and recent studies have found that 4.3%–16% of patients undergoing tumour testing have been found to carry pathogenic (P) or likely pathogenic (LP) germline variants [23]. Given this overlap and the growing relevance of germline variants to treatment selection, greater integration of tumour and germline testing may be advisable [23]. Our workforce requirements model takes this dynamic into account through Pathway 6 (Figure 1), which in the base case assumes that 11.5% of new cancer patients receive tumour testing and P/LP germline mutations are detected in 3% of those patients, which leads to Pathway 6 only accounting for about 1% of the caseload and <1% of FTE requirements. However, while the 11.5% tumour testing rate was calculated based on comprehensive country-wide data from France [24], it reflects practice as of 2017, and the 3% germline variant estimate is from 2015 [25]. If either statistic increases by 2030 (which appears likely based on more recent studies [23]), the impact of germline P/LP variants detected through somatic testing on overall hereditary cancer clinics’ workforce requirements could increase significantly.

On the other hand, the mainstreaming of hereditary cancer-specific testing is being tested in a wide range of clinical settings in oncology [26]. While this could offer a means of addressing the projected increases in the need for hereditary cancer testing, all mainstreaming service delivery models evaluated in the studies reviewed by Bokkers et al. [26] involved referrals of patients harboring P/LP germline variants to genetics clinics for post-test counselling, which limits the magnitude of the impact on GC workforce requirements. Our model suggests that this service delivery model can modestly reduce the need for GC FTEs but would not fully offset the impact of possible expansion in eligibility for hereditary cancer assessment. In addition, mainstreaming appears to lead to dramatically increased uptake of hereditary cancer testing for at least some types of cancer patients [27], which could itself increase the need for GCs for post-test counseling. Moreover, barriers to the widespread implementation of mainstreaming include self-reported inadequacies in knowledge (e.g., 92.7% of recently surveyed oncologists indicated a need for additional training in genomics [28]), as well as lack of time in clinical appointments [26] and concerns about workload [29] expressed by non-genetics healthcare providers (NGHCPs). The former could in principle be addressed by training initiatives as well as clinical decision support tools [30], while the latter could potentially be alleviated by the use of digital decision aids or patient education tools [31]. However, existing genomic and genetic counselling training modules available to NGHCPs [32] have had mixed success [28,33]. In short, mainstreaming is likely to be an important tool for health systems to expand access to hereditary cancer assessment but is unlikely to solve the challenge posed by the growth in eligibility on its own.

In ongoing work, we are expanding our workforce requirements model to incorporate all major clinical areas of genetic medicine [5] and developing a supply model to systematically project the future size of the genetics workforce. We are taking a Canada-wide approach in this research owing to the ease with which GMDs and GCs can move between provinces and substantial existing inter-provincial differences in the provider-to-population ratio [17]. In addition, we are exploring the potential impact on GMD/GC workforce requirements of more speculative changes in the integration of genetics in Canadian health systems, such as population-wide GWS (e.g., Geisinger’s MyCode initiative [34] or the use of GWS for newborn screening [35,36,37]).

One limitation of modeling workforce requirements on a Canada-wide basis is that our model does not consider inter-provincial differences in funded testing eligibility, private pay and self-pay options, mainstreaming practices, or service delivery models. Along with the use of input parameters drawn from non-Canadian sources, and the fact that our model projects need rather than actual referrals or appointments (which in part reflect healthcare-seeking behaviours and barriers to access), our model should not be used alone to project future utilization. However, in previous work we have found that trends in clinical genetics utilization are consistent across high-income countries [5]. Moreover, the careful attention devoted to assessing uncertainty through sensitivity and scenario analyses explores the potential impact of ongoing rapid changes in both the technologies underpinning genetic medicine and scientific knowledge about genetic disease. Finally, we projected total cancer incidence in Canada in 2030 using a simple linear extrapolation of the 2010–2020 trend as reported by the Canadian Cancer Society. While our estimates for 2020 (219,425) and 2030 (265,387) are very similar to projections generated by a more complex micro-simulation model [38] (217,757 in 2020 and 267,041 in 2030) that incorporated Statistics Canada population growth estimates, the recent uptick in international immigration [39], if sustained, may lead to a greater number of new cancer cases than accounted for by our model (and therefore, a greater need for hereditary cancer services). Similarly, while our model does not directly take into account the ethnic composition of the Canadian population, any potential ancestry-based differences in the incidence of cancer are indirectly captured in the overall incidence statistics, and the probability of finding a pathogenic variant does not appear to vary based on ethnicity [40]. While the VUS rate may be higher in patients with ancestry other than European [40], these differences would likely be far outweighed by changes in technology and scientific knowledge over any time period in which substantive changes in overall population composition would unfold.

Overall, our results suggest that, under standard care provision models, growth in the need for hereditary cancer services in Canada by 2030 is likely to, at best, severely strain the capacity of the clinical genetics workforce to meet that need, and, at worst, completely outstrip it. However, our scenario analyses also point to innovations in service delivery that could help GMDs and GCs serve a greater number of patients (Figure 3). Indeed, the only plausible way of meeting the increased need in some scenarios (Figure 2) is to introduce concurrent and substantial changes in service delivery models. While there are significant barriers to widespread mainstreaming, as described above, some of the other innovations (e.g., group counselling) are already being used in some Canadian hereditary cancer clinics [41], while others (e.g., self-administered online decision aids for genetic testing [42,43,44]) have been developed by Canadian groups. Notably, this type of change can be implemented independently by genetics clinics since it does not require significant resources or coordination with other units. However, the successful expansion of mainstreaming to the extent necessary to accommodate the large increases in eligibility for germline testing shown in Figure 2 would require a sustained commitment from both cancer healthcare providers and health systems, as well as additional funding to implement genomics training initiatives aligned with pragmatic service delivery models [45] and to expand the oncology workforce to accommodate increased workloads. In short, successful adaptation to scenarios in which the need for hereditary cancer assessment increases rapidly will require coordinated action at the health system level, which may be most realistic in provinces with higher levels of integration in genetic services and testing [46].

## Figures and Tables

**Table 1:** Base case projections, Canada-wide estimates.

Year	Referrals	Initial Appointments	Results/FU ^1^ Appointments	GMD DPC-FTEs	GMD Headcount	GC DPC- FTEs	GC Headcount	
Deterministic projections								
2020	76,710	52,331	56,961	40.7	52.7	179.2	198.0	
2030	125,904	86,889	93,969	69.7	90.2	295.4	326.4	
Probabilistic projections								
2020 mean (95% CI)	77,295 (53,748 to 108,468)	52,708 (35,925 to 74,484)	57,461 (39,459 to 82,854)	40.9 (31.4 to 53.8)	53.0 (40.6 to 69.6)	180.5 (121.9 to 259.6)	199.5 (134.7 to 286.9)	
2030 mean (95% CI)	126,799 (90,847 to 174,801)	87,457 (61,865 to 1212,329)	94,722 (66,702 to 133,550)	70.0 (55.2 to 90.1)	90.7 (71.4 to 116.6)	297.4 (206.1 to 420.7)	328.6 (227.7 to 464.9)	

^1^ DPC-FTE: Direct patient care full-time equivalent, GMD: medical doctor with expertise in genetics, GC: genetic counsellor, and FU: follow-up. A full description of the model’s assumptions, definitions, and inputs is provided in the Supplementary Materials.

## Data Availability

The data presented in this study are available in this article (and supplementary material).

## Supplementary Materials

The following supporting information can be downloaded at: https://www.mdpi.com/article/10.3390/curroncol30080525/s1, Supplementary Materials and Methods, Table S1: A needs-based planning approach to modeling hereditary cancer services; Table S2: Methods for estimating referral volume for each clinical pathway in hereditary cancer workforce requirements model (base case); Table S3: List of model parameters and probability distributions used for probabilistic sensitivity analysis (base case); Table S4: Aggregation of average time per task in Australian Genomics survey into time per service unit as defined in the GenCOUNSEL workforce requirements model; Table S5: Description of 2030 scenarios, with changes in model as compared to the base case; Table S6: Cancer genetics utilization and projected need in Ontario in 2016; Figure S1: One-way deterministic sensitivity analysis of workforce requirements for MDs with genetics expertise (base case); Figure S2: One-way deterministic sensitivity analysis of genetic counsellor workforce requirements (base case).
