# Supplementary material for: Workforce Implications of Increased Referrals to Hereditary Cancer Services in Canada: A Scenario-Based Analysis
Source: Curr Oncol. 2023 Jul 29;30(8):7241–51. doi: 10.3390/curroncol30080525 (PMC10453026; doi:10.3390/curroncol30080525)
Supplement: Supplementary file 1 [file curroncol-30-00525-s001.zip › curroncol-2494509-supplementary.pdf]

Supplementary Materials and Methods

1. Needs-based planning framework

Our model uses a needs-based planning (NBP) approach, as described by Birch *et al*. (2009) (1). NBP was originally developed as an alternative to demography-based resource planning that could account for changing levels of service required by aging populations that were becoming healthier, as well as changes in the productivity of service providers. The foundation of this approach is to expand from the research question "how many providers are required?" to "how many providers are required to do what, how, for whom, and under what circumstances?" (1). Needs-based projections typically use four types of inputs to determine workforce requirements: specifically, *demographic* and *epidemiological* trends are used to determine the future size of clinically relevant populations in a given jurisdiction, after which the clinically indicated *level of service* for each sub-population and the *productivity* of healthcare providers are used to estimate the size of the workforce necessary to meet the future need implied by the demographic composition and health status of that jurisdiction's population.

Our model for hereditary cancer services in Canada uses the number of new cancer cases diagnosed per year as the main demographic/epidemiological input. The total proportion of these cases and their family members referred for hereditary cancer services are estimated using a range of parameters drawn from the literature that account for clinical guidelines, healthcare provider practice patterns, and patient preferences (Table S1).

2. Volume estimation for each clinical pathway

As described in the main manuscript (Fig. 1), six clinical pathways for hereditary cancer services were defined, and the annual number of patients seen through each pathway in a given jurisdiction were estimated based on the number of new cancer cases in a given year diagnosed in that jurisdiction. We determined the number of new cancer cases using publicly available data from the Canadian Cancer Society. We estimated the future incidence of cancer by applying a linear extrapolation based on the data from the aforementioned source from 2009 to 2020, and checked the plausibility of these estimates by comparing them to published models projecting the future incidence of cancer(2,3). We classified the following cancer types as possibly resulting from hereditary cancer syndromes: breast, ovarian, colorectal, thyroid, endometrial, and pancreatic. In addition, we assumed that 12.5% of cancer cases in Canada diagnosed in individuals under the age of 40 would be flagged for hereditary cancer evaluation even when occurring at primary sites beyond those listed above. Further details on how the volume of referrals for each pathway was estimated in the model's base case are provided in Table S2, and the specific parameter values used and the sources they were drawn from are listed in Table S3.

Table S1. A needs-based planning approach to modeling hereditary cancer services

**NBP input**	**Operationalization in hereditary cancer workforce requirements model**
Demography<br>(population size)	• The volume of new cancer cases as a whole, and broken down by primary site.<br>• These data have a population's demographic structure including age and sex, as well as changes in cancer incidence built into them.
Epidemiology<br>(needs | sub-population)	• Different levels of need are experienced by sub-populations as defined by primary cancer site, age, and whether or not the patient has had cancer or is an unaffected family member of relatives who have had cancer.
Level of service<br>(services | needs)	• The model defines six distinct *care pathways* that include different levels of service according to need.<br>• Referral volumes are assigned to pathways based on the demographic and epidemiological factors described above.
Productivity<br>(provider time | services)	• The amount of provider time required varies based on the different services offered in each care pathway and on the service delivery model used.

**Table S2: Methods for estimating referral volume for each clinical pathway in hereditary cancer workforce requirements model (Base Case)**		
**Pathway**	**Eligible population**	**Estimating the number of referrals**
1. Mainstreaming	Subset of new cancer patients	• The subset of patients entering the mainstreaming pathway were defined as the number of ovarian cancer patients with genetic tests ordered by the their oncologist.<br>• We operationalized this as the proportion of ovarian cancers that are epithelial (90%) times the proportion that occur in people under the age of 70 (80%), resulting in 72% of incident ovarian cancer cases entering this pathway.
2. Genetic counselling only	Family members of cancer patients in previous years	• The starting point is the number of new cases of cancer in the previous year that would be assessed to be possibly hereditary by healthcare providers (total incident cases excluding lung and thyroid cancer and those patients >40 years of age).<br>• Each index case is assumed to have had an average of 3.42 first degree relatives, and 12.6% of them would be expected to receive a high-risk designation.<br>• We also assumed that 56% of them would be informed of their high-risk designation by the index patient and follow through by pursuing a genetics referral.<br>• Finally, we subtract the 70% of these individuals who would seek out self-pay testing (those referrals go to Pathway 3b).
3a. Proband genetic counselling and testing	New cancer patients	• A specific proportion of new, possibly hereditary cancer cases are assumed to be referred to the hereditary cancer program for counseling and testing.<br>• This figure does not include atypical cases (Pathway 5) or mainstreamed cancer types (Pathway 1), which are subtracted out.
3b. Proband genetic counselling and testing	Family members of cancer patients in previous years	• This starts as in Pathway 2 with the relatives of those with possibly hereditary cancers who are at high risk and pursue a referral, but includes those who decide to pursue self-pay genetic testing instead of a counseling-only service.
4. Targeted genetic counselling and testing	Family members of patients with a confirmed	• The number of patients from the other pathways (1, 3, 5 and 6) who test positive for a pathogenic variant is multiplied by the average number of first-degree

	hereditary-cancer-linked variant	relatives that would be informed and the proportion of those that would be expected to follow through with testing.
5. Atypical consultations	Complex cases that require a full consultation with an MD	• This category includes syndromes with extra-oncologic features, or pediatric cases.<br>• Referrals to Pathway 5 are estimated as a proportion (0.171) of the number of new cancer cases estimated to be referred to the hereditary cancer program for counseling and testing (the other 82.9% go to Pathway 3b).<br>• This parameter is drawn from the proportion of initial consultations for hereditary cancer in France that were not for breast, ovarian or digestive tract cancer patients.
6. Somatic testing follow-up	New cases of any type of cancer	• Cancer patients with a germline finding identified through somatic testing.<br>• This was defined as the proportion of new cancer cases that had somatic testing to inform targeted therapy, multiplied by the percentage of individuals undergoing somatic testing who have a germline mutation.

Table S3: List of model parameters and probability distributions used for probabilistic sensitivity analysis (Base Case)

Parameter	Value (mean)	Variance	Distribution assumed in probabilistic sensitivity analysis	Sources (references)
**Base case**				
Proportion of cases which are atypical	0.171	3.71E-07	beta	*Oncogénétique en 2018* (4)
Positive test proportion	0.133	0.000004	beta	*Oncogénétique en 2018* (4)
Additional yield from GWS	1.578947	N/A	FIXED	*Oncogénétique en 2018* (4); Powis et al. (2018) (5)
Positive test proportion for relatives of proband with pathogenic/likely pathogenic variant	0.406	2.12E-05	beta	*Oncogénétique en 2018* (4)
Testing uptake	0.973	0.000626	beta	Calculated from Institut National du Cancer data 2015-2018 (France)<br>*Oncogénétique en 2015* (6)<br>*Oncogénétique en 2016* (7)<br>*Oncogénétique en 2017* (8)<br>*Oncogénétique en 2018* (4)
Proportion of incident cancer cases referred (2030)	0.143	2.5E-07	beta	Linear extrapolation from time-series in *Oncogénétique en 2018* (4)
Proportion of incident cancer cases referred (2020)	0.092	2.12E-07	beta	Linear extrapolation from time-series in *Oncogénétique en 2018* (4)
Proportion of incident cancer cases referred (2017)	0.076	1.76E-07	beta	*Oncogénétique en 2018* (4)

Number of first-degree family members per positive proband	3.42	0.14	gamma	Levin, 2017 (9); Menko, 2019 (10) (Wagner, Patenaude from this review); Fehniger, 2013(11); weighted average
Number of relatives informed per positive proband	4.59	3.46	gamma	Menko, 2019 (10) (Blandy, McGivern, Wagner, Sermijn, Patenaude, Finley, Fehniger); weighted average
Proportion of high-risk relatives (2017)	0.126	N/A	FIXED	Han (2017)(12)
- (proportion in 2020)	0.126	N/A	FIXED	Han, 2017(12)
- (projected in 2030)	0.146	N/A	FIXED	Calculated from Institut National du Cancer data 2015-2018 (4,6–8). This value makes the growth from 2020-2030 consistent with the growth trend seen between 2015-2018
High risk family members who pursue referral	0.56	0.01	beta	Levin, 2017 (9), Menko review (10) (uptake of testing #s, not specifically consultation; Blandy, Finlay, Aktan-Collan, Bodd, Cody, Sanz, Aktan-Collan, Barrow, Seppala); weighted average
Referred family members who pursue private pay testing	0.7	0.04	beta	Weymann, 2017 (13); Van Bebber, 2007 (14)
Proportion of individuals in the pathway who use a decision aid	0.89	0.000724	beta	Adam, 2019 (15)
Total number of hours per annual FTE (37.5hrs/week, for 48 weeks per year)	1800	N/A	FIXED	Calculation
Proportion of referrals to genetics declined by genetics clinic	0.168	0.000	beta	Benjamin et al. (2015) (16)
Proportion of patients with an accepted referral who decline to make an appointment	0.148	0.000	beta	Benjamin et al. (2015) (16)

Proportion of patients with an appointment who do not attend	0.087	0.000	beta	Benjamin et al. (2015) (16)
Proportion of ovarian cancers that are epithelial (90%) X proportion under the age of 70 yo (80%)	0.72	N/A	FIXED	GTEOC study, UK based report (2017) (17)
Average proportion of cancer cases that are diagnosed <40 in Canada	0.049	0.000	beta	Canadian Cancer Society, 2017 (18)
Proportion of individuals undergoing somatic testing who have an underlying germline mutation	0.03	0.000	beta	Jones et al, 2015 (19)
Number of patients who had a NGS somatic test that led to targeted therapy	0.115	0.000	beta	Calculated from INC, 2017 (20)
Proportion of individuals who have testing who would be seen for a results appointment	1	N/A	FIXED	Base Case assumption - all have results appointment
Proportion of individuals who need an additional follow-up appointment	0.16	N/A	FIXED	Hereditary Cancer Program, BC Cancer Agency. Calculated from HCP data (for every 6 results appt. they have 1 follow-up appt. slot)
Calculated times per service unit for MDs (complex cases) (minutes) [Australian times – converted in model to Canadian context by multiplying by 0.83]				
Triage	0	N/A	Fixed	Various (see section 3.1 and Table S4 below)
Initial appointment	128	19.9	Gamma	Various (see section 3.1 and Table S4 below)
Test coordination	51	13.0	Gamma	Various (see section 3.1 and Table S4 below)
Results appointment	288	32.1	Gamma	Various (see section 3.1 and Table S4 below)
Additional appointment	87	17.5	Gamma	Various (see section 3.1 and Table S4 below)
Calculated times per service unit for GCs (minutes) [Australian times – converted in model to Canadian context by multiplying by 0.83]				
Triage	48	4.9	Gamma	Various (see section 3.1 and Table S4 below)
Initial appointment	144	14.3	Gamma	Various (see section 3.1 and Table S4 below)

Test coordination	43	6.3	Gamma	Various (see section 3.1 and Table S4 below)
Results appointment	207	17.7	Gamma	Various (see section 3.1 and Table S4 below)
Additional appointment	102	12.8	Gamma	Various (see section 3.1 and Table S4 below)

3. Service units

Provider tasks have been defined for each clinical pathway (Figure 1 in the main manuscript) as a combination of five service units: 1) triage process, 2) initial appointment, 3) test coordination, 4) results appointment, and 5) additional follow-up appointments. The appointment service units include both the time required for non-patient-facing related activities (e.g., chart review, administrative tasks, etc...) as well as face-to-face appointment time. The combination of service units is unique to a given pathway depending on what is required for patients in that clinical context.

3.1 Time estimates for service units

Our main data source for the time spent by genetic counsellors and clinical geneticists on specific tasks was a workforce survey conducted by the Australian Genomics Health Alliance (Australian Genomics) in 2017 (21). Table C-6 of the Technical Report provides the average time and standard deviation spent by 163 GCs and 79 clinical geneticists (CGs) on a set of specific clinical tasks performed in the context of clinical care for genetics patients. While the average time per task was also reported separately for different clinical areas (e.g., prenatal genetics, pediatric genetics, etc...), average times did not vary consistently across clinical contexts, so we used the overall averages for each task as inputs.

Task-based time estimates from the Australian Genomics survey were aggregated into time estimates to deliver each service unit in the model as shown in Table S3 below. However, genetic service delivery in Australia has differences in reimbursement, scope of practice, and regulation, as compared to Canada, which make it likely that the times estimated based on Australian Genomics data cannot be directly transferred to a Canadian context due to, for example, different average caseloads per genetic counsellor. Indeed, the Australian Genomics survey reported that genetic counsellors saw 23 patients per month on average, and given that the average full-time equivalent (FTE) worked was 0.82, this equates to a 1.0 FTE caseload of 28 patients per month. In Canada, the Canadian Association of Genetic Counsellors' (CAGC) 2016 Professional Status Survey indicated that Canadian GCs saw an average of 313 patients per year (or 26.1 per month), but only 67% of GCs worked full-time. If we estimate that the remaining 33% on average had a 0.5 FTE contract, the average caseload per 1.0 FTE would be 31.1 patients per month. This is similar to the 30.6 face-to-face patient encounters per month reported for Ontario GCs by Shugar et al. (2017) (22), though this study did not indicate average FTE for the respondents. Assuming a similar full-time proportion as in the CAGC survey, this suggests an average of 33.9 patients seen per 1.0 FTE in Canada per month ((26+30.6)/2/0.835), as compared to an estimated 28.0 patients seen per 1.0 FTE in Australia, suggesting a slightly higher average caseload per counsellor in Canada. As such, we multiplied the time per service unit estimates in Table S4 by the ratio of estimated caseloads (0.828) to adjust the time estimates to a Canadian context. Similar caseload estimates for clinical geneticists were not available, so we used the GC ratio to adjust MD time estimates from the Australian to Canadian context.

Note that time units used in the model were adjusted in some pathways to capture the differences in roles and scope of practice between GCs and MDs for different patient types. For appointments in pathways where the GC is likely to be the lead care provider under current service delivery models, the GC time was estimated from the Australian Genomics survey above to reflect the time required both for appointment time and patient related activities (Table S4). However, the MD time was set at 30 minutes for patient-interacting service units (initial, results, and additional follow-up appointments) (BC Cancer Agency; personal communication) and 0 minutes for patient-related activity

Table S4: Aggregation of average time per task in Australian Genomics survey into time per service unit as defined in the GenCOUNSEL workforce requirements model

Australian Genomics Survey				Time per service unit (Australian context) [subject to caseload weighting conversion to Canadian context]				
**Task label**	**Task name**	**GC time, minutes - mean (sd)**	**MD time, minutes - mean (sd)**	**Service unit**	**Tasks (GC)**	**GC time, minutes – mean (sd)**	**Tasks (GMD)**	**GMD time, minutes – mean (sd)**
A	Initial intake call	24 (17)	36 (39)	***1. Triage process***	A + (G_2 * 0.5)	48 (28)	-	-
B	Initial consultation	49 (17)	60 (23)	Intake call	A	24 (19)	-	-
C	Test coordination	43 (32)	51 (32)	Admin paperwork	G_2 * 0.5	24 (21)	-	-
D	Research and interpreting results	105 (28)	201 (35)	***2. Initial appointment***	B + F + G_1 + (G_2 * 0.5)	144 (49)	B + F + G_1 + G_2	128 (40)
D_1	Literature and database searches re: counselling	26 (27)	51 (33)	Appointment	B	49 (17)	B	60 (23)
D_2	Clinical meetings and consultations	31 (31)	34 (29)	Patient-related activities	F + G_1	71 (40)	F + G_1	52 (30)
D_3	Analyzing test results, incl. Literature and database searches	25 (27)	50 (33)	Admin paperwork	G_2 * 0.5	24 (21)	G_2	16 (12)
D_4	Other	23 (28)	66 (46)	***3. Test coordination***	C	43 (32)	C	51 (32)
E	Further consults	31 (22)	35 (22)	***4. Results appointment***	E + D + F + G_1	207 (54)	E + D + F + G_1	288 (51)
F	Follow-up activities	39 (29)	37 (28)	Appointment	E	31 (22)	E	35 (22)
G	Administration	80 (35)	31 (12)	Patient-related activities	D + F + G_1	176 (49)	D + F + G_1	253 (46)
G_1	Data entry, appointments, etc...	32 (28)	15 (11)	***5. Additional follow-up appointment***	E+ F + G_1	102 (46)	E + F + G_1	87 (37)
G_2	Other	48 (42)	16 (12)	Appointment	E	31 (22)	E	35 (22)
				Patient-related activities	F + G_1	71 (40)	F + G_1	52 (30)

service units (triage, test coordination). This MD time captures their roles in patient-related activities such as case review, case management, and reviewing case documentation. In Canada, GCs are often able to lead care provision for encounters that do not require protected medical acts (e.g., physical exam, new diagnoses, etc…) and for which the focus is genetic counseling. Consequently, in our model GCs are the lead care providers for Pathway 1, Pathway 2, Pathway 3, Pathway 4, and Pathway 6 (see Table S2 for a full description of the pathways), with MDs providing clinical oversight. In contrast, in cases that involve tasks that are more medical in nature and involve protected medical acts the MD is classified as the lead care provider. These appointments are captured in Pathway 5. To account for differences in roles in these appointments, the times have been adjusted. The MD time for Pathway 5 is therefore adapted from the Australian Genomics survey as described in Table S4 and it is assumed that the time GCs spend on these appointments is half of what they spend on the same tasks when they are the lead provider for that encounter.

3.2 FTE and headcount calculations

To enhance interpretability of our model estimates for readers, we used the following procedure to convert the direct patient care full-time equivalent (DPC-FTE) workforce requirements estimates generated by our model to estimated headcounts (i.e., the number of healthcare providers (HCPs) needed to provide that patient care capacity). The calculation proceeds in two steps.

First, DPC-FTEs are converted to total standard FTEs required by adding in the time necessary for HCPs to perform essential non-clinical duties, such as supervision, professional development, and administration. For GCs involved in direct patient care, the typical proportion of weekly working hours spent on direct patient care is 84% (23), while for MDs it is 58% (24). The definition of a standard full-time equivalent (FTE) in our workforce requirements model is the total compensated time an individual works in one standard work week, which we assume to be equal to 37.5 hours (with 48 weeks of work per year, for a total of 1,800 hours worked per year). If we were to assume that all HCPs do in fact work 37.5 hours/week, then the number of estimated total FTEs required would be equivalent to the headcount required. However, some proportion of HCPs work part-time, and in reality both GCs and MDs work more than 37.5 hours/week on average. Recent workforce surveys indicate that 90 percent of genetic counsellors have full-time positions, working 41.6 hours per week on average (25), and part-time genetic counsellors work 71% of full-time hours on average (29.5 hours). Thus 100 GCs working in clinical positions will have a capacity of 90.5 DPC-FTEs as defined by our model:

100 GCs involved in direct patient care *

(((0.9*41.6 + 0.1 *29.5) [total number of hours worked per week for FT and PT workers]

/37.5)) [as a multiple of total FTEs]

*0.84) [multiplied by the proportion of total time used for direct patient care]

= 90.5 GC direct patient care FTEs

We can therefore multiply the GC DPC-FTE estimates generated by the model by 1.105 (100/90.5) to estimate the number of GCs working in clinical positions that would be required to maintain that capacity. Similarly, for GMDs, including medical geneticists, who work an average of 50 hours per week (24) and for whom we assume that 100% work full-time, we can estimate that:

100 MDs specialized in genetics *

50 [total number of hours worked per week]

/37.5 [as a multiple of standard clinical FTEs]

*0.58) [multiplied by the proportion of total time used for direct patient care]

= 77.3 GMD direct patient care FTEs

To infer required GMD headcount we can therefore *multiply GMD DPC-FTE estimates by 1.294* (100/77.3). As an alternative estimate for GCs, we performed the same calculations assuming actual hours worked per week were 37.5 rather than 41.6, which results in an estimated capacity of 81.6 DPC-FTEs for every 100 GCs involved in direct patient care (100*((0.9*37.5 + 0.1* 26.6)/37.5)*0.84).

4. Scenarios

Table S5 provides a list of all the scenarios evaluated in this study. Our previous Delphi panel survey of Canadian clinical genetics experts (26) pointed to two potential trends that could substantially increase the need for genetic counselling for hereditary cancer in Canada by 2030. Namely, 72% of respondents believed it was likely that "the guidelines for considering who is eligible for germline genetic testing for hereditary cancer will be expanded to include all types of cancer" and 61% thought it was likely that "tumor testing will be performed using genome-wide sequencing (GWS; i.e. exome or genome sequencing) instead of another genetic test". Respondents' average estimate of the "percentage of individuals with any type of cancer who will receive germline genetic testing for hereditary cancer variants" in 2030 was 32% (SD=9.3; range 20% to 50%), which we use to operationalize Scenario 1 below.

In the Australian Genomics survey described above, Australian GCs and GMDs were asked to report time per task separately for GWS vs. non-GWS cases. Total time spent for GWS patients was an average of 519 minutes (SD=26) for GCs and 548 minutes (SD=27) for GMDs, as compared to 371 minutes (SD=27) and 454 minutes (SD=28), respectively, for non-GWS patients. This implies that a GWS patient on average takes 40% longer for GCs and 21% longer for GMDs ((21); Table D-2 compared with Table C-6). While some evidence in the literature suggests that GWS may result in an increased diagnostic yield for hereditary cancer variants over gene panels (27–29), these were small studies, and a head-to-head randomized clinical trial was unable to establish a diagnostic yield advantage for exome sequencing (30). In part this likely reflects the frequent updating of gene panels with newly discovered pathogenic variants, and means that any comparative advantage is likely to be small and of limited durability for a given variant. We therefore assume no advantage in our GWS Substitution scenario (#2 below).

5. Sensitivity analyses

To explore the range and sources of uncertainty associated with our model's estimated workforce requirements, we conducted both deterministic and probabilistic sensitivity analyses for the *Base Case* of the model. For the probabilistic sensitivity analysis, probabilistic distributions were defined for a 27 of the 31 non-jurisdiction-specific parameters used in the *Base Case*, with beta distributions used for proportions and probabilities and gamma distributions use for time parameters (see Table S3). We then conducted a 1000-run Monte-Carlo simulation in which parameter values were randomly sampled from the 27 distributions in each run and DPC-FTE estimates were calculated for each run. The mean DPC-FTEs over the 1000 runs were used as the point estimates for workforce required, while 95% confidence intervals were estimated using the 2.5th and 97.5th percentiles of the resulting DPC-FTE distributions.

**Table S5 – Description of 2030 scenarios, with changes in model as compared to the Base Case**		
**Scenario**	**Description**	**Operationalization**
Scenario 1:<br>Expanded eligibility	Expansion in the eligibility for germline testing for individuals with a new case of cancer grows faster in 2020-2030 than in 2010-2020.	The proportion of new cancer cases referred for hereditary cancer genetic services is 32% (Scenario 1b) vs. 14.3% (Base Case, 2030)<br><br>Scenarios 1a and 1c explore the range of the Delphi panel estimates where the proportion of new cancer cases referred for genetic services ranged from 20% to 50%, respectively.<br><br>Scenario 1d considers the impact of an expansion of referrals to 32% of new cancer cases, but with this trend accompanied by increased mainstreaming, with all new breast cancer cases having a genetic predisposition test ordered by their treating oncologist/surgeon, and only patients with positive tests being referred to a genetics clinic (Pathway 1).
Scenario 2:<br>GWS test substitution	Gene panels currently used in hereditary cancer testing are all replaced with GWS (exome or genome sequencing). Technology used for targeted testing confirming known variants is unchanged.	Time per patient for probands undergoing genetic testing through the cancer genetics clinic (Pathways 3, 5) increases by 40% for GCs and 21% for GMDs.
Scenarios 3a-3d:<br>Innovation in genetic counseling service delivery	Evaluates the impact on workforce requirements of the following…	In general, the average time per service unit required per patient changes to the following values:
	3a) using an ***online decision aid*** for pre-test counselling,	3a) Assumes that 89% of patients would use an online decision aid as part of pre-test counselling if it was available (15). Of these, 65% of patients would not receive any genetic counselling, while 35% of patients would have a pre-test appointment with a GC, but these would be 50% shorter than standard appointments. For those patients who do not receive any pre-test counselling, GC time for patient-related activities would be reduced by 75% and appointment time would be 0. This scenario applies to Pathways 3, 4, 5. GMD time in Pathways 3 and 4 would be unchanged (since it is already assumed that GMD involvement is limited), while in Pathway 5 GMD

		pre-test time would be reduced in the same way as for GCs in Scenarios 3 and 4. For GCs in Pathway 5, the baseline time per appointment is already reduced by 50% to account for increased MD involvement. As a result, GC times for patients who receive the decision aid are further reduced by the same proportion by which the use of the decision aid reduces full GC appointments in the other pathways (by 17% when the patient has a GC appointment after using the decision aid, and by 71% when the patient declines a pre-test genetic counselling appointment).
	3b) returning ***negative test results*** by letter,	3b) Results appointments and PRA for non-mainstreamed patients with negative test results are assumed to be 15 minutes per provider. This scenario applies to Pathways 3, 4, 5.
	3c) use of ***genetic assistants*** (Gas) to perform more administrative tasks to allow genetic counsellors to practice at top-of-scope, and	3c) The PRA time for genetic counsellors (including triage and test coordination) is reduced by 65% (27). There is no impact on appointment times. This does not apply to atypical cases and the addition of a GA does not impact GMD time. This scenario applies to Pathways 1, 2, 3, 4, and 6.
	3d) the use of ***group pre-test counseling*** appointments	3d) The GC time required for initial appointments is assumed to be 0.49 of the time needed for individual appointments and 10 minutes per patient for GMDs (28) .This scenario applies to non-mainstreamed GC-led appointments (Pathways 2, 3, 4).
	3e) ***expanded mainstreaming*** of germline genetic testing for cancer patients	3e) Germline testing is ordered by treating oncologist and/or surgeon for all new breast cancer cases(29–32) and those patients with positive tests flow into Pathway 1. No patients with breast cancer are referred through Pathway 3.

Deterministic sensitivity analysis

In addition, to see which parameters had the largest effect on the DPC-FTE estimates, we performed a one-way deterministic sensitivity analysis in which each parameter was separately varied +/- 25% while holding the other parameter values constant. The resulting DPC-FTE results are presented in the tornado plot below.

Figure S1: One-way deterministic sensitivity analysis of workforce requirements for MDs with genetics expertise (Base Case)

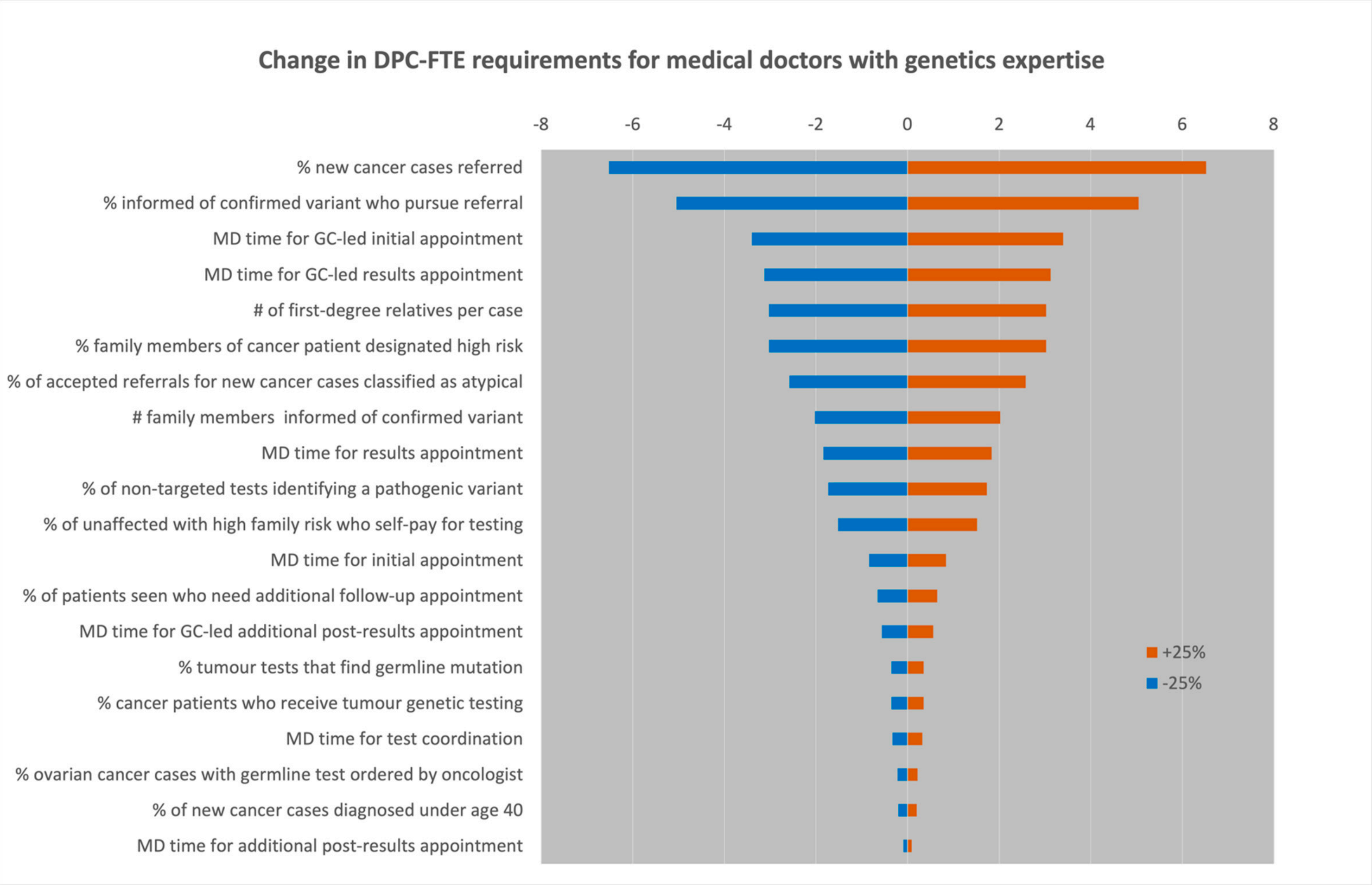

Figure S2: One-way deterministic sensitivity analysis of genetic counsellor workforce requirements (Base Case)

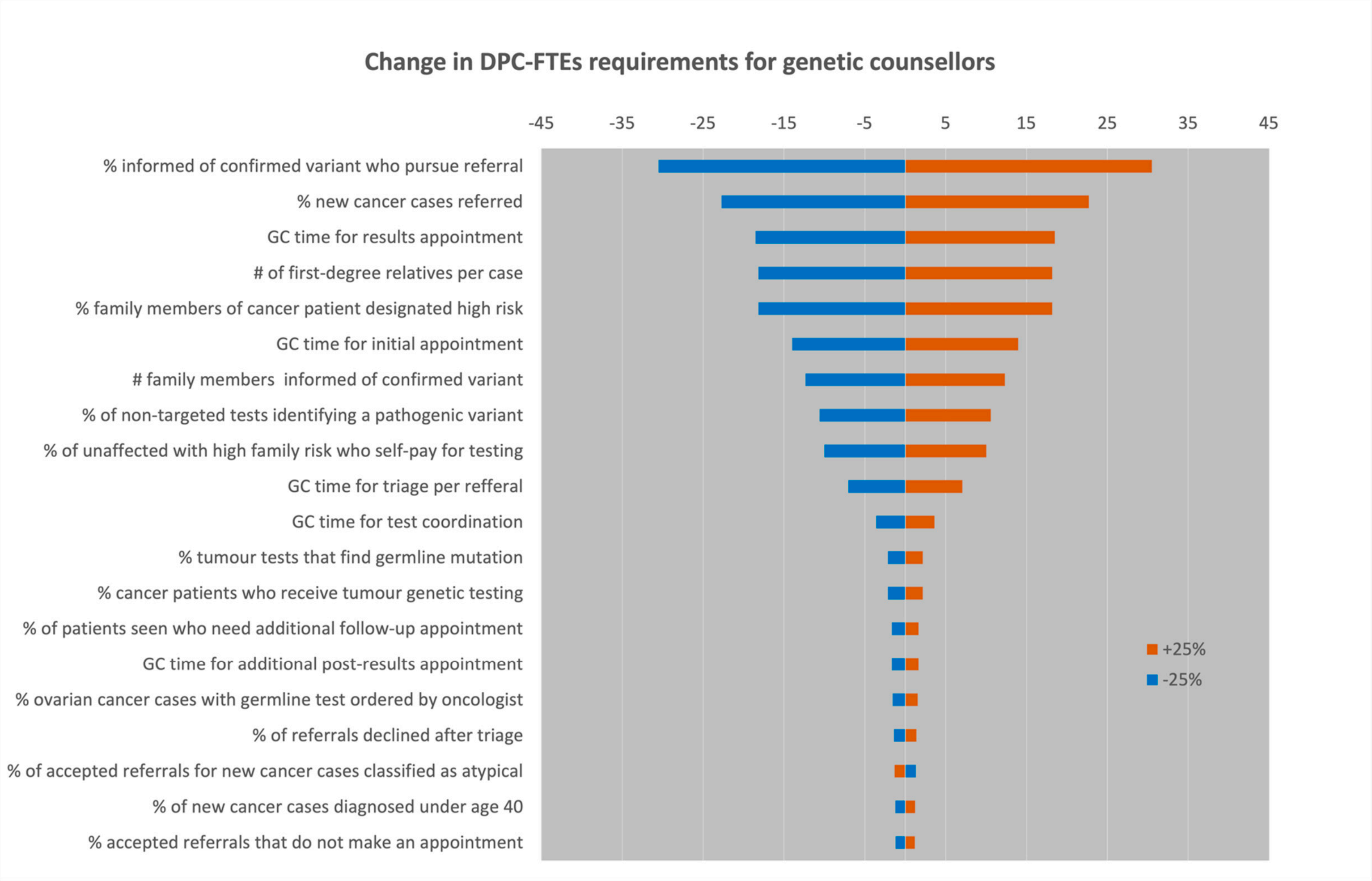

6. Comparison with Cancer Care Ontario needs-based workforce analysis

We sought to validate our approach by comparing our model's estimates using 2016 Ontario cancer incidence data as an input with both reported utilization and a needs-based workforce requirements projection published by Cancer Care Ontario in 2018 (33). Using the number of new cancer cases in Ontario in 2016 as an input, our Base Case model estimated a referral volume of 26,879 cases for 2016 and a requirement of 62.8 GC DTC-FTEs to provide clinical care to those patients (Table S6). While the estimated number of referrals was 45% greater than the actual number of referrals actually received, it was only 4% higher than the *need* estimated by Cancer Care Ontario's modeling (despite our model using a significantly different structure and no Ontario utilization data as an input) (33), and the estimated number of patients seen per GC FTE in our model was within 10% of the actual reported ratio in Ontario. This level of agreement provides with Cancer Care Ontario's own needs-based model provides cross-validation of our model output, and lends face validity to our Canada-wide estimates.

Table S6: Cancer genetics utilization and projected need in Ontario in 2016

	Referrals	*Initial appointments*	*Results/follow-up appointments*	*GC DPC-FTEs (total)*	*Appts/DPC-FTE*
Actual utilization, Cancer Care Ontario (33)	18,084	13,883	n/a	43.4	319
Need, Cancer Care Ontario (33)	25,810	n/a	n/a	78.1	331
Need, GenCOUNSEL model	26,879	18,277	n/a	62.8	291

References

1. Birch S, Kephart G, Murphy GT, O'Brien-Pallas L, Alder R, MacKenzie A. Health human resources planning and the production of health: development of an extended analytical framework for needs-based health human resources planning. J Public Health Manag Pract. 2009;15(6 Suppl):56–61.

2. Canadian Cancer Society's Advisory Committee on Cancer Statistics. Canadian Cancer Statistics 2015 Special topic: Predictions of the future burden of cancer in Canada. Toronto, ON; 2015.

3. Poirier AE, Ruan Y, Walter SD, Franco EL, Villeneuve PJ, King WD, et al. The future burden of cancer in Canada: Long-term cancer incidence projections 2013–2042. Cancer Epidemiol. 2019 Apr 1;59:199–207.

4. Institut National du Cancer. Oncogenetique en 2018. 2020.

5. Powis Z, Espenschied CR, LaDuca H, Hagman KD, Paudyal T, Li S, et al. Clinical germline diagnostic exome sequencing for hereditary cancer: Findings within novel candidate genes are prevalent. Cancer Genet. 2018 Aug 1;224–225:12–20.

6. Institut National du Cancer. Oncogenetique en 2015/ Consultations et laboratoires, collection appui a la decison. 2017.

7. Institut National du Cancer. Oncogenetique en 2016/ consultations, laboratoires et suivi [Internet]. 2017 [cited 2022 Jul 3]. Available from: e-cancer.fr

8. Institut National du Cancer. Oncogenetique en 2017/consultations et laboratoires. 2019.

9. Levin T, Mæhle L. Uptake of genetic counseling, genetic testing and surveillance in hereditary malignant melanoma (CDKN2A) in Norway. Fam Cancer. 2017;16(2):257–65.

10. Menko FH, ter Stege JA, van der Kolk LE, Jeanson KN, Schats W, Moha DA, et al. The uptake of presymptomatic genetic testing in hereditary breast-ovarian cancer and Lynch syndrome: a systematic review of the literature and implications for clinical practice. Vol. 18, Familial Cancer. Springer Netherlands; 2019. p. 127–35.

11. Fehniger J, Lin F, Beattie MS, Joseph G, Kaplan C. Family communication of BRCA1/2 results and family uptake of BRCA1/2 testing in a diverse population of BRCA1/2 carriers. J Genet Couns. 2013;22(5):603–12.

12. Han X, Jemal A. Recent patterns in genetic testing for breast and ovarian cancer risk in the U.S. Am J Prev Med. 2017 Oct 1;53(4):504–7.

13. Weymann D, Veenstra DL, Jarvik GP, Regier DA. Patient preferences for massively parallel sequencing genetic testing of colorectal cancer risk: a discrete choice experiment. European Journal of Human Genetics. 2018 Sep 1;26(9):1257–65.

14. van Bebber SL, Liang SY, Phillips KA, Marshall D, Walsh J, Kulin N. Valuing personalized medicine: Willingness to pay for genetic testing for colorectal cancer risk. Per Med. 2007 Aug;4(3):341–50.

15. Adam S, Birch PH, Coe RR, Bansback N, Jones AL, Connolly MB, et al. Assessing an interactive online tool to support parents' genomic testing decisions. J Genet Couns. 2019;28(1):10–7.

16. Benjamin C, Houghton C, Foo C, Edgar C, Mannion G, Birch J, et al. A prospective cohort study assessing clinical referral management & workforce allocation within a UK regional medical genetics service. European Journal of Human Genetics [Internet]. 2015;23(8):996–1003. Available from: http://dx.doi.org/10.1038/ejhg.2015.33

17. Cameron L, Plaskocinska I, Kroese M, Tischkowitz M. Delivering improved access to genetic testing in epithelial ovarian cancer. 2016.

18. Canadian Cancer Society's Advisory Committee on Cancer Statistics. Canadian Cancer Statistics 2017 Special topic: Pancreatic cancer [Internet]. Toronto, ON; 2017 Jun [cited 2022 Jul 4]. Available from: cancer.ca/Canadian-Cancer-Statistics-2017-EN.pdf

19. Jones S, Anagnostou V, Lytle K, Parpart-Li S, Nesselbush M, Riley DR, et al. Personalized genomic analyses for cancer mutation discovery and interpretation. Sci Transl Med [Internet]. 2015 Apr 15;7(283):283ra53. Available from: https://www.science.org

20. Institut National du Cancer. Les tests de genetique somatique. 2017.

21. Nisselle A, Macciocca I, Mckenzie F. AUSTRALIAN GENOMICS GENOMIC WORKFORCE , EDUCATION & ETHICS Technical Report Project 2 : Professional Status Survey of Genetic Counsellors and Clinical Geneticists. (May 2018):1–73. Available from: https://www.australiangenomics.org.au/publications/professional-status-survey-of-genetic-counsellors-and-clinical-geneticists/

22. Shugar AL, Quercia N, Trevors C, Rabideau MM, Ahmed S. Risk for patient harm in Canadian genetic counseling practice: It's time to consider regulation. J Genet Couns. 2017;26(1):93–104.

23. Attard CA, Carmany EP, Trepanier AM. Genetic counselor workflow study: The times are they a-changin'? J Genet Couns. 2019;28(1):130–40.

24. Jenkins BD, Fischer CG, Polito CA, Maiese DR, Keehn AS, Lyon M, et al. The 2019 US medical genetics workforce: a focus on clinical genetics. Genetics in Medicine. 2021 Aug 1;23(8):1458–64.

25. National Society of Genetic Counselors. 2021 Professional Status Survey: Work Environment. 2021.

26. Borle K, Kopac N, Dragojlovic N, Rodriguez Llorian E, Friedman JM, Elliott AM, et al. Where is genetic medicine headed? Exploring the perspectives of Canadian genetic professionals on future trends using the Delphi method. European Journal of Human Genetics [Internet]. 2022; Available from: https://doi.org/10.1038/s41431-021-01017-2

27. Pirzadeh-Miller S, Robinson LS, Read P, Ross TS. Genetic Counseling Assistants: An integral piece of the evolving genetic counseling service delivery model. J Genet Couns. 2017;26(4):716–27.

28. Hynes J, MacMillan A, Fernandez S, Jacob K, Carter S, Predham S, et al. Group plus "mini" individual pre-test genetic counselling sessions for hereditary cancer shorten provider time and improve patient satisfaction. Hered Cancer Clin Pract. 2020 Feb 19;18(1).

29. Bell KA, Kim R, Aronson M, Gillies B, Ali Awan A, Chun K, et al. Development of a comprehensive approach to adult hereditary cancer testing in Ontario. J Med Genet [Internet]. 2022 Dec 23

[cited 2023 Jan 26];jmg-2022-108945. Available from: https://jmg.bmj.com/lookup/doi/10.1136/jmg-2022-108945

30. Culver JO, Freiberg Y, Ricker C, Comeaux JG, Chang EY, Banerjee V, et al. Integration of Universal Germline Genetic Testing for All New Breast Cancer Patients. Ann Surg Oncol. 2022 Feb 1;30(2):1017–25.

31. Whitworth PW, Beitsch PD, Patel R, Rosen B, Compagnoni G, Baron PL, et al. Clinical Utility of Universal Germline Genetic Testing for Patients with Breast Cancer. JAMA Netw Open. 2022 Sep 22;5(9):E2232787.

32. Yin K, Chai TS, Wooters M, Shannon KM, Hughes KS. Mainstreamed genetic testing of patients with breast cancer: Experience from a single surgeon's practice in a large U.S. academic center. Journal of Clinical Oncology. 2022 Jun 1;40(16_suppl):10577–10577.

33. Cancer Care Ontario. Recommendation Report for Ontario's Clinical Genetic Services [Internet]. 2018. Available from: https://www.cancercareontario.ca/sites/ccocancercare/files/assets/ClinicalGeneticServicesRecommendationReport.pdf
